# Advanced glycation end products regulate anabolic and catabolic activities *via* NLRP3‐inflammasome activation in human nucleus pulposus cells

**DOI:** 10.1111/jcmm.13067

**Published:** 2017-02-22

**Authors:** Yu Song, Yan Wang, Yukun Zhang, Wen Geng, Wei Liu, Yong Gao, Shuai Li, Kun Wang, Xinghuo Wu, Liang Kang, Cao Yang

**Affiliations:** ^1^ Department of OrthopedicsUnion HospitalTongji Medical CollegeHuazhong University of Science and TechnologyWuhanChina; ^2^ Department of Physical EducationChina University of GeosciencesWuhanChina; ^3^ China Medical UniversityShenyangChina; ^4^ Department of OrthopedicsFirst Hospital of WuhanWuhanChina

**Keywords:** intervertebral disc degeneration, advanced glycation end products, human nucleus pulposus cells, IL‐1β, NLRP3 inflammasome, mitochondrial damage, calcium

## Abstract

Intervertebral disc degeneration is widely recognized as a cause of lower back pain, neurological dysfunction and other musculoskeletal disorders. The major inflammatory cytokine IL‐1β is associated with intervertebral disc degeneration; however, the molecular mechanisms that drive IL‐1β production in the intervertebral disc, especially in nucleus pulposus (NP) cells, are unknown. In some tissues, advanced glycation end products (AGEs), which accumulate in NP tissues and promote its degeneration, increase oxidative stress and IL‐1β secretion, resulting in disorders, such as obesity, diabetes mellitus and ageing. It remains unclear whether AGEs exhibit similar effects in NP cells. In this study, we observed significant activation of the NLRP3 inflammasome in NP tissues obtained from patients with degenerative disc disease compared to that with idiopathic scoliosis according to results detected by Western blot and immunofluorescence. Using NP cells established from healthy tissues, our *in vitro* study revealed that AGEs induced an inflammatory response in NP cells and a degenerative phenotype in a NLRP3‐inflammasome‐dependent manner related to the receptor for AGEs (RAGE)/NF‐κB pathway and mitochondrial damage induced by mitochondrial reactive oxygen species (mtROS) generation, mitochondrial permeability transition pore (mPTP) activation and calcium mobilization. Among these signals, both RAGE and mitochondrial damage primed NLRP3 and pro‐IL‐1β activation as upstream signals of NF‐κB activity, whereas mitochondrial damage was critical for the assembly of inflammasome components. These results revealed that accumulation of AGEs in NP tissue may initiate inflammation‐related degeneration of the intervertebral disc *via* activation of the NLRP3 inflammasome.

## Introduction

Intervertebral disc (IVD) degeneration and other pathological changes in the spine are major causes of back pain and a substantial drain on medical resources worldwide 1, 2, 3. Unfortunately, the IVD frequently acquires a degenerative phenotype in adulthood, and secondary structural degeneration occurs to varying degrees over the age of 30 4, 5, although lower back pain does not always manifest immediately. The major proinflammatory cytokine IL‐1β accelerates disc degeneration by promoting an inflammatory response, stimulating degradation of the extracellular matrix, and inducing angiogenesis and neo‐innervation 6, 7, 8, 9. Notably, the IVD, especially the NP tissue, is shielded from the host immune system by lack of vascularization and structural isolation. Nevertheless, NP cells are capable of behaving as competent phagocytes and producing proinflammatory cytokines 8, 10. However, the molecular mechanisms associated with promoting IL‐1β production in NP cells remain unclear.

IL‐1β maturation and secretion require the inflammasome, a multi‐protein complex generally composed of immature caspase‐1, adaptor protein apoptosis‐associated speck‐like (ASC) protein and one of the nucleotide‐binding oligomerization domain (NOD)‐like receptors 11, 12, 13. The NOD‐like receptor assembles the inflammasome when activated by an array of microbial and endogenous stimuli and subsequently undergoes proximity‐induced autocatalysis to cleave caspase‐1 into the active subunits p10 and p20, which, in turn, cleave pro‐IL‐1β to release mature IL‐1β. Of the NOD‐like receptors, NLRP3 is the major sensor that mediates both sterile inflammatory and antimicrobial responses 12, 13. Therefore, it has gained increasing attention in pathophysiological studies of human disease. Consequently, most endogenous crystals, such as urate, calcium pyrophosphate dehydrate and β‐amyloid, were confirmed to activate the NLRP3 inflammasome and trigger chronic inflammation 12, 13.

AGEs are formed by non‐enzymatic glycation of macromolecules, also known as the Maillard reaction. These compounds accumulate in long‐lived proteins during ageing and degeneration and contribute to stiffness, brittleness and biomechanical alterations in collagen‐rich tissues 14. Recent studies showed that these compounds also accumulate in the IVD, especially in diabetic individuals, and are associated with up‐regulation of extracellular matrix proteinases, inflammation and degeneration 15, 16, 17, 18. Therefore, we suggested that AGEs promote IVD degeneration by activating the NLRP3 inflammasome and inducing IL‐1β secretion in NP cells.

To test this hypothesis, we analysed the distribution of NLRP3 inflammasome in human NP tissues and its correlation with IVD degeneration. Additionally, we established primary *in vitro* cultures of human NP cells to investigate the mechanisms driving AGEs‐induced inflammatory and degenerative response.

## Materials and methods

### Collection of NP tissues

Experimental protocols were approved by the Ethics Committee of Tongji Medical College, Huazhong University of Science and Technology. Degenerative NP tissues from 15 males and 20 females, aged 32–64 years (mean: 48.6 years), were collected from patients undergoing surgery due to degenerative disc disease (DDD). Healthy tissues from patients with no DDD were also collected from two males and three females, aged 15–21 years (mean: 17.8 years), who underwent surgery for idiopathic scoliosis (IS). Specimens were immediately sectioned for use in various experiments. One section was immediately fixed in 4% buffered formaldehyde (pH 7.4) for eventual histological analysis. A second section was immediately immersed in RNAlater (Invitrogen, Carlsbad, CA, USA) and frozen in liquid nitrogen for use in protein and RNA analysis. A third section was immediately immersed in phosphate‐buffered saline (PBS) for cell isolation.

### Isolation and culture of human NP cells

NP cells were isolated from healthy tissues as described previously 15, plated and expanded for 3 weeks at 37°C and 5% CO_2_ in Dulbecco's modified Eagle medium containing 15% foetal bovine serum (Gibco, Waltham, MA, USA) and 1% penicillin/streptomycin (Invitrogen). The culture medium was replaced twice every week, except that primary cells were allowed more time (6.7 ± 1.4 days) to adhere prior to the first change of medium. Cells from the second passage were used in further experiments.

### *In vitro* experiments in human NP cells and reagents

NP cell cultures were serum‐starved for 12 hrs and exposed to 100 μg/ml bovine serum albumin (BSA) for 48 hrs or AGEs‐BSA (Merck Millipore, Darmstadt, Germany) for 0, 12, 24, 36 and 48 hrs. In some experiments, cells were pre‐treated with a neutralizing antireceptor for AGE (RAGE) antibody (100 μg/ml; R&D Systems, Minneapolis, MN, USA) for 1 hr, TPCA‐1 (1 μM; Selleck Chemicals, Houston, TX, USA) for 2 hrs, MitoTEMPO (50 μM; Abcam, Cambridge, UK) for 2 hrs, BAPTA‐AM (20 μM; Selleck Chemicals) for 1 hr and cyclosporin A (20 μM; Selleck Chemicals) for 2 hrs and then co‐cultured with BSA or AGEs‐BSA. To knock down NLRP3, cells were transfected for 48 hrs with 100 nM NLRP3 siRNA or scrambled siRNA (GenePharma, Shanghai, China) in Lipofectamine 2000 (Invitrogen) and immediately stimulated with BSA or AGEs‐BSA in the presence or absence of a caspase‐1 inhibitor (VX‐765; Selleck Chemicals), recombinant human IL‐1β or IL‐1Ra (R&D Systems).

Protein expression in lysate was analysed by Western blot using antibodies specific for NLRP3 (Invitrogen), ASC, RAGE (Abcam), phosphorylated IKKα/β (p‐IKKα/β, S180/S181), IKKα, phosphorylated IκBα (p‐IκBα, S32/S36), IκBα, phosphorylated NFκB‐p65 (p‐NFκB‐p65, S536), NFκB‐p65, pro‐caspase‐1, caspase‐1, pro‐IL‐1β and IL‐1β (Cell Signaling Technology, Danvers, MA, USA). Fluo‐3 AM for intercellular calcium analysis, DCFH‐DA for intercellular reactive oxygen species (ROS) assay, MitoSOX for mtROS assay and JC‐1 for mitochondrial membrane‐potential (MMP) assay were purchased from Beyotime (Shanghai, China). A mPTP assay kit was obtained from BioVision (Milpitas, CA, USA).

### Immunofluorescence microscopy

NP tissues were sectioned at 4 μm, deparaffinized in xylene, rehydrated through graded ethanol and blocked with endogenous peroxidase in hydrogen peroxide, and the antigens were retrieved with citrate buffer. Sections were probed at 4**°**C overnight with primary antibodies (1:200) against NLRP3 and cleaved‐caspase‐1 in 2% BSA and PBS, washed thoroughly and labelled for 40 min. at 37**°**C with secondary antibody (1:200; Vector, Burlingame, CA, USA).

By contrast, cultured human NP cells were mounted on coverslips, washed with PBS, fixed with 4% paraformaldehyde for 15 min. at 37**°**C, permeabilized with 0.5% (v/v) Triton X‐100 for 20 min. and blocked with 1% (w/v) goat serum albumin for 30 min. Samples were then washed, probed at 4**°**C overnight with antibodies against NLRP3, ASC or RAGE and labelled for 40 min. with a secondary antibody. Additionally, for mitochondrial localization, Mitotracker Green buffer (Beyotime) was incubated with cultured human NP cells for 40 min. before fixation.

Finally, nuclei were co‐stained for 5 min. with 0.1 g/ml 4′,6‐diamidino‐2‐phenylindole, and samples were washed and imaged with a fluorescence microscope. The expression of protein was quantified by integrated optical density (IOD) with Image‐Pro Plus image analysis system.

### Reverse transcription and quantitative real‐time PCR

Total RNA was extracted with Trizol reagent (Invitrogen) from NP tissues and cultured cells, reverse‐transcribed and amplified by PCR. Briefly, 1 μg total RNA was reverse‐transcribed using an All‐in‐One first‐strand cDNA synthesis kit (GeneCopoeia, Guangzhou, China), and 1 μl of the resulting cDNA was amplified by PCR with an ABI7900 Eco real‐time PCR system (Illumina, San Diego, CA, USA) using 200 μM specific primers and 10 μl 2 × SYBR Green/Fluorescein qPCR master mix. PCR reactions were incubated at 50**°**C for 2 min. and 95**°**C for 10 min., followed by 40 cycles at 95**°**C for 30 sec. and 60**°**C for 30 sec. Results were normalized against β‐actin according to the 2^−ΔΔCt^ method. Primers were previously validated as capable of amplifying target genes. Primer sequences were as follows: 5′‐TTCGGAGATTGTGGTTGGG‐3′ and 5′‐AGGGCGTTGTCACTCAGGT‐3′ for NLRP3, 5′‐ ATGGCTTATTACAGTGGCA ‐3′ and 5′‐ TGTAGTGGTGGTCGGAGA ‐3′ for IL‐1β, 5′‐GATGCGCAAGCCCAGGTGTG‐3′ and 5′‐GCCAATTTCATGAGCAGCAACGA‐3′ for matrix metalloproteinase 3, 5′‐TCAGGAAACCAGGTCTGGAG‐3′ and 5′‐TGACGCGAACAATACGGTTA‐3′ for matrix metalloproteinase 13, 5′‐AATTCCGACCTCGTCATCAG‐3′ and 5′‐GCCTGGATAACCTCTGTG‐3′ for COL2A1 and 5′‐AGCGAGCATCCCCCAAAGTT‐3′ and 5′‐GGGCACGAAGGCTCATCATT‐3′ for β‐actin.

### Western blot

Tissue or cellular protein was lysed and extracted with cold lysis buffer (Beyotime). Protein (40 μg) from each sample was subjected to 4–20% precast polyacrylamide gel (Bio‐Rad) electrophoresis and transferred to nitrocellulose membranes (Bio‐Rad, Hercules, CA, USA). The primary antibody dilution rates vary from 1:500 to 1:1000, for NLRP3, ASC, pro‐caspase‐1, caspase‐1, pro‐IL‐1β, IL‐1β, RAGE, phosphorylated IKKα/β, IKKα, phosphorylated IκBα, IκBα, phosphorylated NFκB‐p65 and NFκB‐p65 detection, followed by 1:3000 dilution of goat anti‐rabbit or antimouse HRP‐labelled antibody (Bio‐Rad). β‐actin was used as the protein loading control.

### Measurement of caspase‐1 activity

The FAM‐FLICA Caspase‐1 Assay Kit was used to detect active caspase‐1 as per the manufacturer's instructions. Briefly, cultured human NP cells were mounted on coverslips and washed with PBS. Then, the active caspase‐1 was assessed by the FLICA reagent FAM‐YVAD‐FMK, which could irreversibly and specifically bind to activated caspase‐1.

### Enzyme‐linked immunosorbent assay

Media were collected from cell cultures at 0, 12, 24, 36 and 48 hrs and analysed for IL‐1β using enzyme‐linked immunosorbent assay kits (Abcam).

### Flow cytometry

Fluo‐3 AM, DCFH‐DA, MitoSOX, JC‐1 and a mPTP assay kit were used according to the manufacturer's instructions. Mean fluorescence intensity was then measured with a FACSCalibur flow cytometer (BD Biosciences, San Jose, CA, USA).

### Statistical analysis

Data were analysed in SPSS 17.0 (IBM, Chicago, IL, USA) and are reported as mean ± S.D. of three independent experiments. An unpaired *t*‐test was used to compare healthy and degenerated tissues, while nonparametric linear regression was used to assess the correlation of NLRP3 and cleaved‐caspase‐1 with Pfirrmann grade 19, a measure of IVD degeneration. Student's *t*‐test was used to compare *in vitro* treatment groups. A *P* < 0.05 was considered statistically significant.

## Results

### The NLRP3 inflammasome was related to IVD degeneration

**Figure 1:**
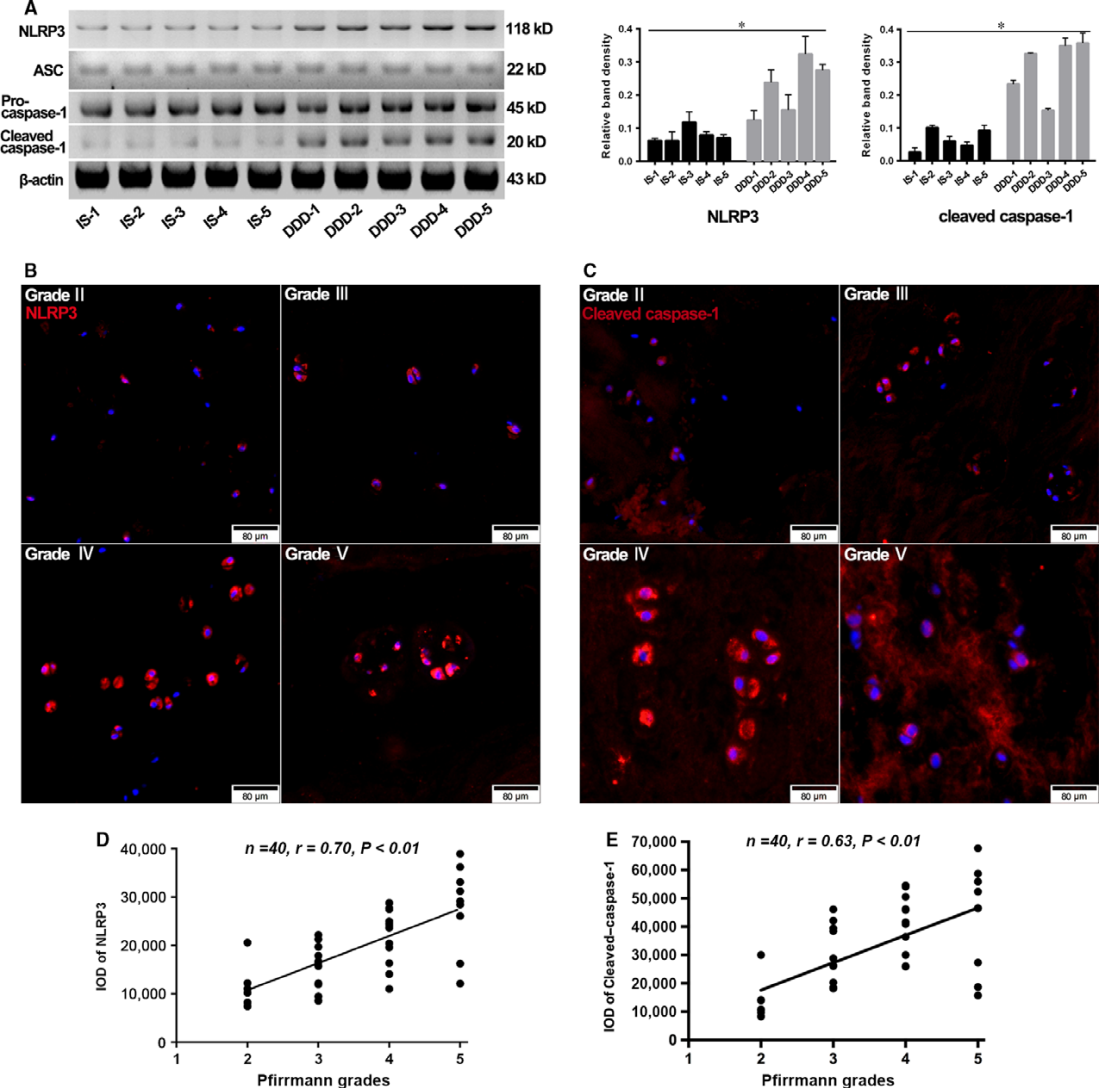
The human intervertebral disc (IVD) degeneration positively correlated with NLRP3‐inflammasome activation. (**A**) Representative Western blot assay (left) and quantitation (right) of the level of NLRP3‐inflammasome components in nuclear pulposus (NP) tissues collected from patients undergoing surgery due to idiopathic scoliosis (IS) or degenerative disc disease (DDD). Data were presented as mean ± S.D. (*n* = 3). *P < 0.05 *versus* IS group. (**B** and **C**) Representative fluorescent images of 40 NP tissues divided into Grade II, Grade III, Grade IV and Grade V by Pfirrmann grades. Red fluorescence indicated NLRP3 and cleaved‐caspase‐1 signals, respectively. Cell nuclei are stained by DAPI. (**D** and **E**) Correlation analysis between NLRP3 and cleaved‐caspase‐1 and disc degenerative grade. The level of NLRP3 and cleaved‐caspase‐1 in each grade was characterized by corresponding IODs analysed with Image‐Pro Plus image analysis system. Data were presented as mean (*n* = 3).

We assessed the NLRP3‐inflammasome components by five degenerative and five healthy NP tissue specimens. In comparison with healthy NP tissues, activation of the NLRP3 inflammasome was enhanced in degenerative tissues, as indicated by more abundant expression of NLRP3 and by increased cleavage of caspase‐1, although ASC and pro‐caspase‐1 were expressed at comparable levels (Fig. 1A). Furthermore, to study the relationship of NLRP3 inflammasome to degenerative NP progress, the level of NLRP3 and cleaved‐caspase‐1 was detected by immunofluorescence microscopy in 40 NP tissues (Fig. 1B and C), which included 7 at Grade II, 12 at Grade III, 12 at Grade IV and 9 at Grade V by Pfirrmann grades 19. As shown in Figure 1D and E, NLRP3 and cleaved‐caspase‐1 showed positive correlation with disc degeneration.

### AGEs‐BSA activated the NLRP3 inflammasome and elicited IL‐1β production in human NP cells

**Figure 2:**
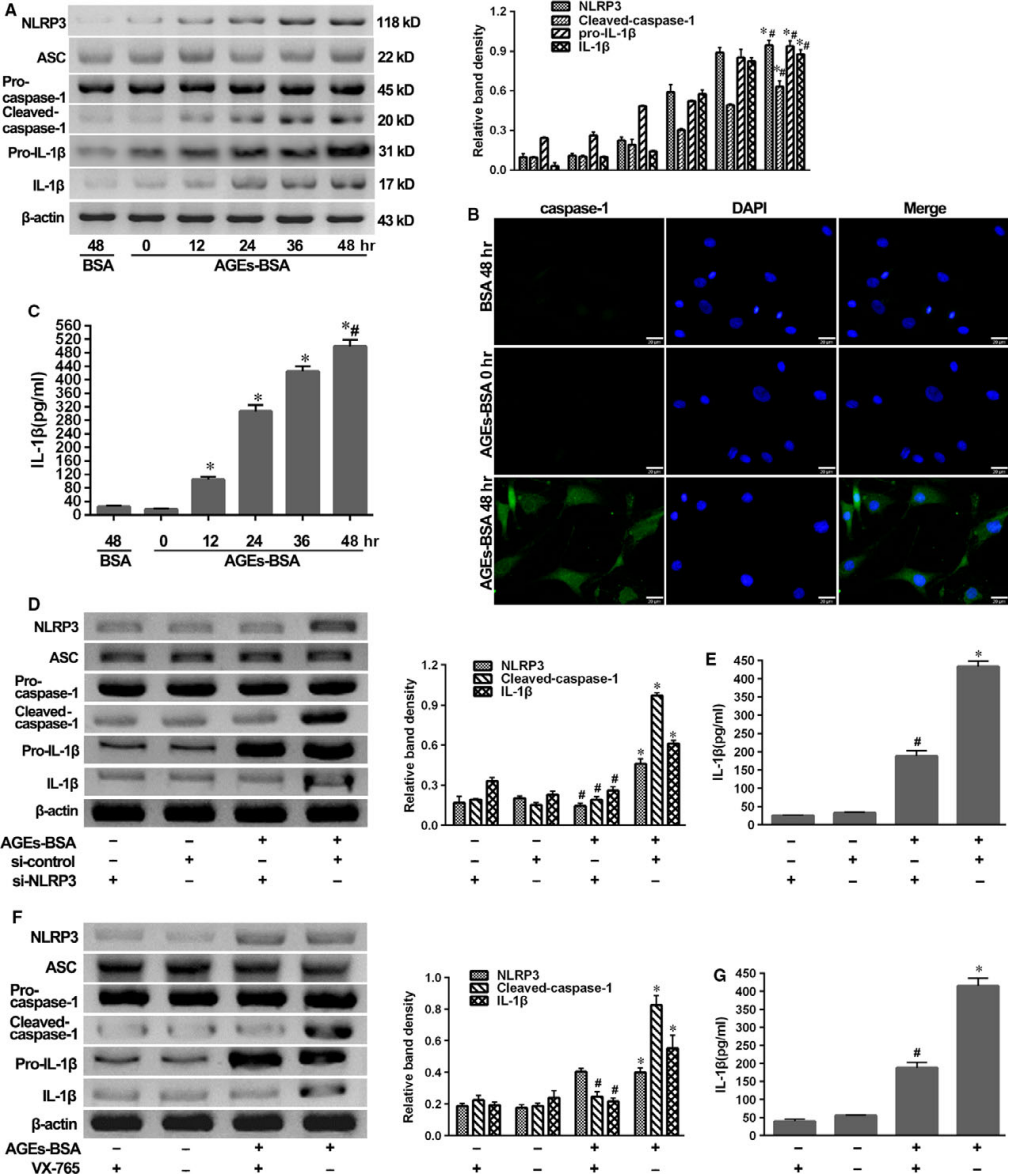
AGEs‐BSA induced the NLRP3‐inflammasome activation and secondary IL‐1β secretion. NP cells were isolated from IS patients. (**A**) Representative Western blot assay (left) and quantitation (right) of the expression profiles of NLRP3‐inflammasome components, pro‐IL‐1β and IL‐1β in NP cells after treatment with AGEs‐BSA (100 μg/ml) for 0–48 hrs or BSA (100 μg/ml) for 48 hrs. Data were presented as mean ± S.D. (*n* = 3). *P < 0.05 *versus* BSA (48 hrs). #*P* < 0.05 *versus* 0 hr. (**B** and **C**) Representative fluorescent images of caspase‐1 activity (**B**) and enzyme‐linked immunosorbent assay for the secretion profiles of IL‐1β (**C**) in NP cells or supernatant as described above. Data were presented as mean ± S.D. (*n* = 3). *P < 0.05 *versus* BSA (48 hrs). #*P* < 0.05 *versus* 0 hr. (**D** and **F**) Representative Western blot assay (left) and quantitation (right) of the expression profiles of NLRP3‐inflammasome components, pro‐IL‐1β and IL‐1β and (**E** and **G**) enzyme‐linked immunosorbent assay for the secretion profiles of IL‐1β in NP cells or supernatant induced by AGEs‐BSA for 48 hrs in the presence or absence of si‐NLRP3 transfection (**D** and **E**) or caspase‐1 inhibitor VX‐765 (**F** and **G**). Data were presented as mean ± S.D. (*n* = 3). *P < 0.05 *versus* si‐control, #*P* < 0.05 *versus* AGEs‐BSA + si‐control.

To determine whether AGEs‐BSA could induce IL‐1β production in human NP cells *via* NLRP3‐inflammasome activation, we examined the status of the NLRP3 inflammasome and IL‐1β in human NP cells stimulated with AGEs‐BSA for 0–48 hrs or BSA for 48 hrs as control. Western blot results showed that AGEs‐BSA induced time‐dependent increases in NLRP3, cleaved‐caspase‐1, pro‐IL‐1β and IL‐1β levels, but not those of ASC or pro‐caspase‐1 (Fig. 2A). Following treatment of NP cells for 48 hrs, AGEs‐BSA significantly increased their levels as compared with those observed in BSA controls. Similarly, enhanced activity of caspase‐1 (Fig. 2B) and the increased secretion of mature IL‐1β into culture media (Fig. 2C) were detected following AGEs‐BSA treatment.

To further confirm that AGE‐induced IL‐1β production was dependent upon the NLRP3 inflammasome, NP cells were exposed to AGEs‐BSA in the presence of NLRP3 knockdown by si‐NLRP3 or the specific caspase‐1 inhibitor VX‐765. We observed that si‐NLRP3 achieved significant NLRP3 knockdown and then prevented the processing of pro‐caspase‐1 and pro‐IL‐1β cleavage (Fig. 2D) and the section of IL‐1β (Fig. 2E). In addition, inhibition of caspase‐1 activity by VX‐765 blocked the cleavage of pro‐caspase‐1 and pro‐IL‐1β and the release of IL‐1β in response to AGEs‐BSA treatment (Fig. 2F and G).

### Activation of the NLRP3 inflammasome was associated with AGEs‐induced anabolic and catabolic genes expression in human NP cells

**Figure 3:**
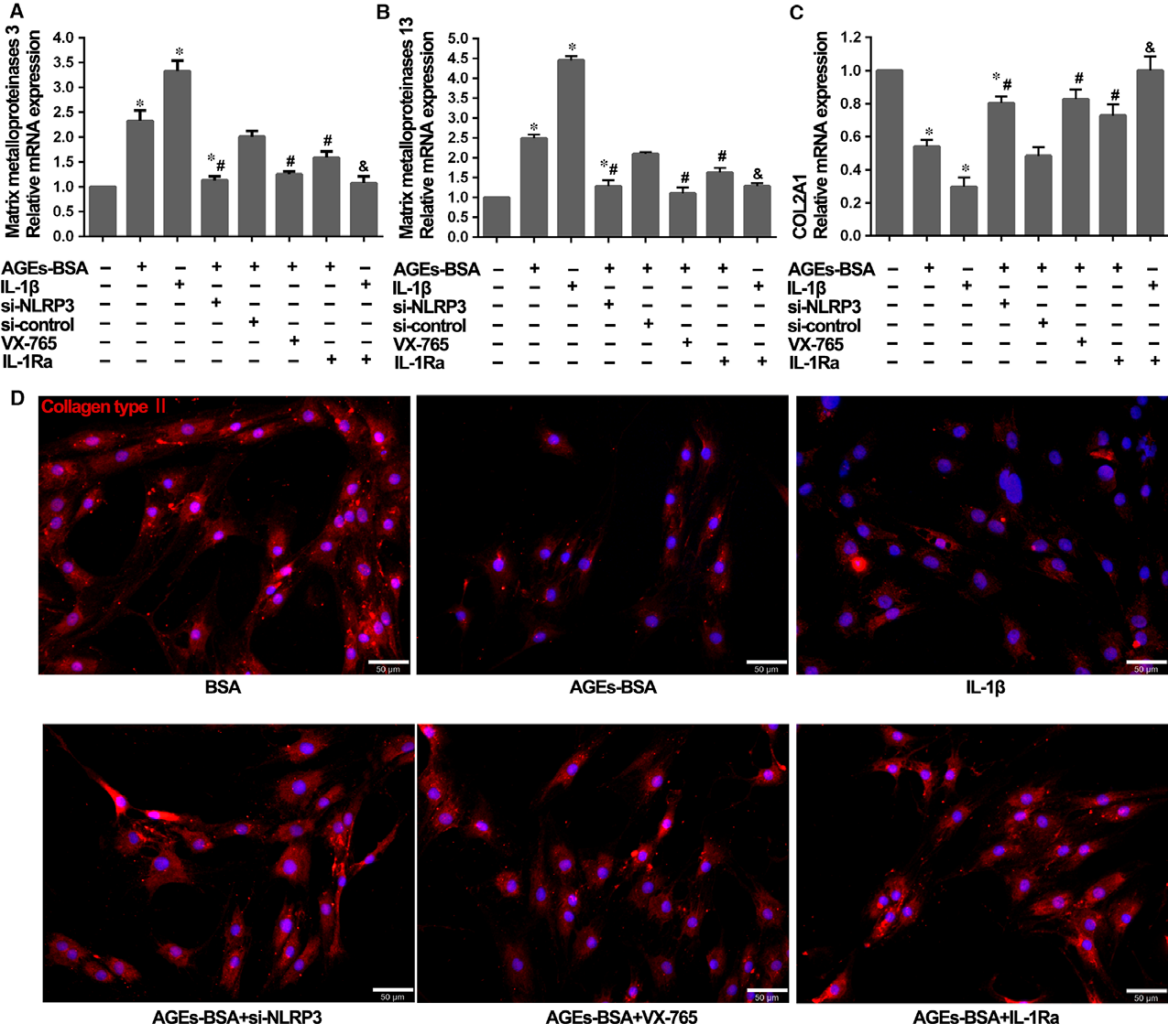
The effects of NLRP3‐inflammasome activation and IL‐1β secretion on the degenerative phenotype of NP cells. (**A, B** and **C**) Quantitative real‐time PCR for the transcript expression levels of matrix metalloproteinases 3, matrix metalloproteinases 13 and COL2A1 in NP cells after treatment with AGEs‐BSA (100 μg/ml) or recombinant human IL‐1β (1 ng/ml) for 48 hrs, combined with si‐NLRP3, VX‐765 and IL‐1Ra or not. Data were presented as mean ± S.D. (*n* = 3). *P < 0.05 *versus* control, #*P* < 0.05 *versus* AGEs‐BSA + si‐control, &*P* < 0.05 *versus* IL‐1β. (**D**) Representative fluorescent images of collagen type II expression in NP cells treated with single or combined reagents as shown in figure. Red fluorescence indicated collagen type II signals. Cell nuclei are stained by DAPI.

To determine whether activation of the NLRP3 inflammasome promoted NP degeneration, cultured cells were exposed for 48 hrs to 100 μg/ml AGEs‐BSA or 1 ng/ml recombinant human IL‐1β. Quantitative real‐time PCR showed that AGEs‐BSA or IL‐1β treatment significantly up‐regulated matrix metalloproteinase 3 and matrix metalloproteinase 13 expression (Fig. 3A and B), concomitant with down‐regulated COL2A1, responsible for collagen type II expression (Fig. 3C). NLRP3 knockdown and inhibition of caspase‐1 by VX‐765 or IL‐1β‐receptor antagonist IL‐Ra could significantly reverse these degenerative effects induced by AGEs‐BSA (Fig. 3A–C). In addition, the immunofluorescence staining of collagen type II confirmed that AGEs‐BSA or IL‐1β treatment inhibited the expression of collagen type II, which could be rescued by NLRP3 knockdown, VX‐765 or IL‐Ra. Together, these results indicated that the blockage of collagen type II expression induced by AGEs‐BSA was dependent on the activation of NLRP3 inflammasome (Fig. 3D).

### AGEs‐BSA induced RAGE/NF‐κB signalling to prime NLRP3 and pro‐IL‐1β

**Figure 4:**
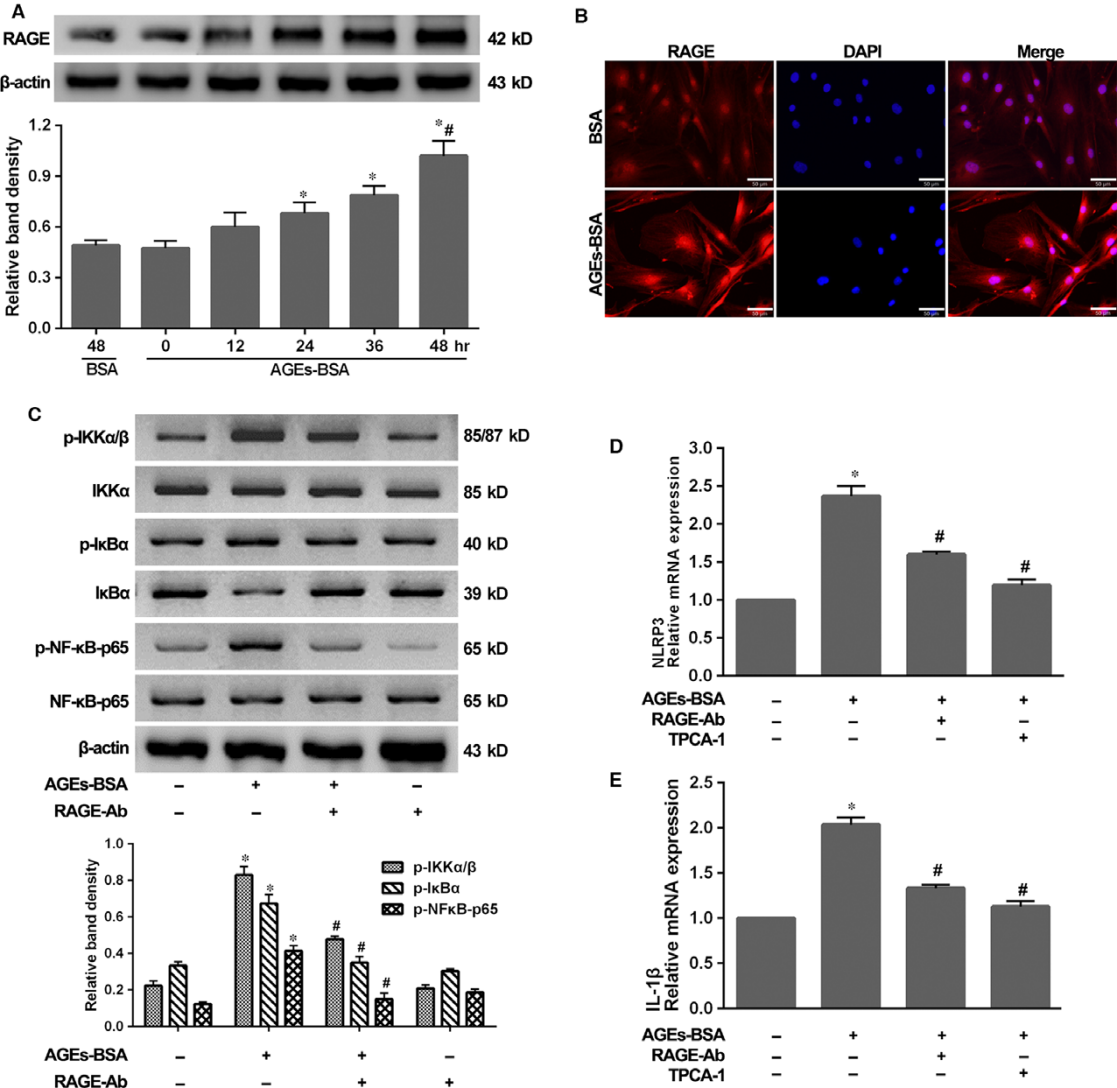
AGEs‐BSA‐induced RAGE/NF‐κB activation was responsible for NLRP3 and IL‐1β transcription. (**A**) Representative Western blot assay (upper) and quantitation (bottom) of RAGE level in NP cells as described in Figure 2A. Data were presented as mean ± S.D. (*n* = 3). *P < 0.05 *versus* 0 hr. #*P* < 0.05 *versus* BSA (48 hrs). (**B**) Representative fluorescent images of RAGE in NP cells treated with BSA or AGEs‐BSA for 48 hrs. Red fluorescence indicated RAGE signals. Cell nuclei are stained by DAPI. (**C**) Representative Western blot assay (upper) and quantitation (bottom) showing phosphorylated (p‐) and total protein levels of IKKα/β, IκBα and NF‐κB‐p65, and (**D** and **E**) quantitative real‐time PCR for the transcript expression levels of NLRP3 and IL‐1β in NP cells induced by AGEs‐BSA for 48 hrs, single or combined with RAGE‐Ab or TPCA‐1. Data were presented as mean ± S.D. (*n* = 3). *P < 0.05 *versus* control, #*P* < 0.05 *versus* AGEs‐BSA.

RAGE is expressed at low levels under basal conditions, but is up‐regulated in response to diverse pathological events. Here, we observed that RAGE was detectable soon after NP cells were stimulated with AGEs‐BSA and accumulated over time (Fig. 4A and B). To further investigate whether RAGE participated in the regulation of NF‐κB signalling and further influenced NLRP3‐inflammasome activation, we treated NP cells with AGEs‐BSA in the presence or absence of the neutralizing anti‐RAGE antibody (RAGE‐Ab) or the IKK‐2 inhibitor (TPCA‐1). As shown in Figure 4C, RAGE‐Ab could significantly block the NF‐κB signalling induced by AGEs‐BSA. Furthermore, both inhibition of RAGE by RAGE‐Ab and blockage of NF‐κB phosphorylation by TPCA‐1 could significantly block NLRP3 and pro‐IL‐1β expression in response to AGEs‐BSA treatment (Fig. 4D and E). These results indicated that RAGE and NF‐κB mediated the expression of NLRP3 and pro‐IL‐1β in response to AGEs.

### ROS generation and calcium mobilization induced by AGEs‐BSA

**Figure 5:**
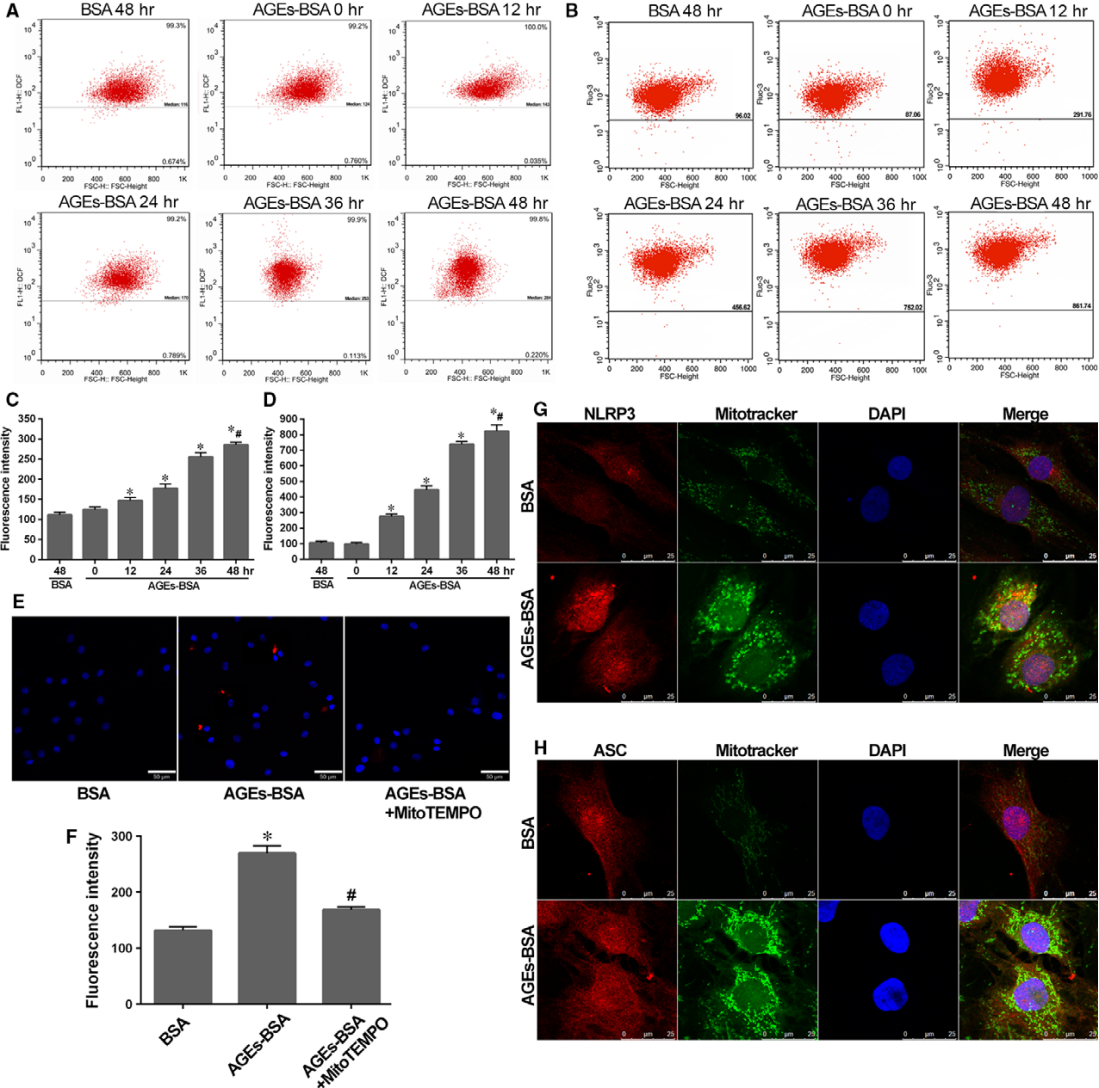
AGEs‐BSA promoted robust ROS generation and calcium mobilization in NP cells. The enhanced perinuclear localization of mitochondria and NLRP3/ASC indicated the association with NLRP3‐inflammasome activation. (**A** and **B**) Representative dot plot by flow cytometry analysis after labelling with fluorescent probe DCFH‐DA for ROS or Fluo‐3 AM for calcium in NP cells as described in Figure 2A. (**C** and **D**) Quantitative analysis of fluorescence intensity showed the corresponding ROS and calcium levels in NP cells. Data were presented as mean ± S.D. (*n* = 3). *P < 0.05 *versus* BSA (48 hrs); #*P* < 0.05 *versus* 0 hr. (**E**) Representative fluorescent images of mitochondrial ROS generation marked by MitoSOX (red), a mitochondrial ROS dye, in NP cells induced by AGEs‐BSA or pre‐treated with MitoTEMPO. Cell nuclei are stained by DAPI. (**F**) Quantitative analysis of fluorescence intensity of NP cells as described above. Data were presented as mean ± S.D. (*n* = 3), *P < 0.05 *versus* BSA; #*P* < 0.05 *versus* AGEs‐BSA. (**G** and **H**) NP cells showed an enhanced perinuclear localization of mitochondria and NLRP3/ASC in NP cells induced by AGEs‐BSA for 48 hrs. Red and green fluorescence indicated NLRP3/ASC and mitochondria signals, respectively. Cell nuclei are stained by DAPI.

As for NLRP3‐inflammasome activation, ROS generation and calcium mobilization were approved to play vital roles 20, 21. Here, the ROS and cytosolic calcium level were, respectively, probed by DCFH‐DA and Fluo‐3 AM and detected using flow cytometer. We observed a time‐dependent increase in intercellular ROS and calcium levels, showing an increasing fluorescence intensity. After 48 hrs of culture, NP cells treated with AGEs‐BSA showed significantly increased ROS and calcium level compared to that with BSA (Fig. 5A–D). To further define the source of ROS, we marked and scavenged mtROS, respectively, with MitoSOX and MitoTEMPO. As shown in Figure 5E, increased mtROS level and positive cells were significantly detected by fluorescence scope in group with AGEs‐BSA treatment compared to that with BSA. Moreover, MitoTEMPO significantly reduced both mtROS and total ROS levels (Fig. 5E and F), which indicated that mitochondria acted as main ROS resource in NP cells induced by AGEs‐BSA.

Mitochondria and NLRP3/ASC protein are assumed as different localized distributions under resting or stimulated conditions 22. To confirm the critical role of mitochondria in NLRP3‐inflammasome activation, we analysed the location of NLRP3/ASC and mitochondria in NP cells with treatment of AGEs‐BSA or BSA. Mitochondria presented as a long, filamentous morphology in BSA treatment. By contrast, in the presence of AGEs‐BSA, mitochondria co‐localized in the perinuclear regions with NLRP3 and an increased presence of NLRP3 level according to, respectively, fluorescence staining (Fig. 5G). The relocated tendency as above was not seen for ASC, which showed an extensive distribution in cells in both conditions (Fig. 5H).

### MMP decrease, mPTP activation and vicious circle with ROS and calcium

**Figure 6:**
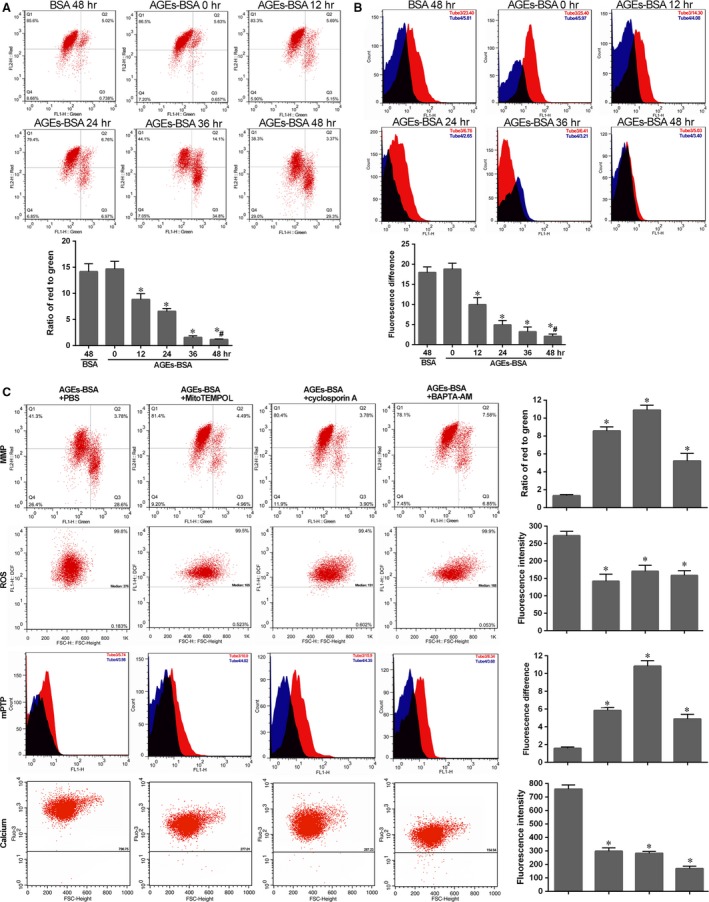
Mitochondrial damage in NP cells induced by AGEs‐BSA and its interaction with ROS generation and calcium mobilization. Mitochondrial membrane potential (**A**) and mitochondrial permeability transition pore (**B**) were detected by flow cytometry analysis. (**A**) Representative scatter plots (top) and quantitative analysis of the ration of red to green (bottom) in NP cells as described in Figure 2A after labelling with fluorescent probe JC‐1. FL1‐H: green (JC‐1 monomers); FL2‐H: red (JC‐1 aggregates). (**B**) Representative peak chart (top) and quantitative analysis of the difference between Tube 3 and Tube 4 (bottom) in NP cells after labelling with fluorescent probe MPTP staining dye. FL1‐H: fluorescence intensity by fluorescent probe MPTP staining dye. NP cells in Tube 3 (red) were exposed to CoCl_2,_ and that in Tube 4 (blue) were exposed to CoCl_2_ and ionomycin. The black part was overlapping. Data were presented as mean ± S.D. (*n* = 3). *P < 0.05 *versus* 0 hr. #*P* < 0.05 *versus* BSA (48 hrs). (**C**) MMP, ROS, mPTP and calcium levels were analysed in NP cells induced by AGEs‐BSA for 48 hrs, respectively, pre‐treated with MitoTEMPO (a specific mtROS scavenger), cyclosporin A (a blocker of mPTP) and BAPTA‐AM (calcium chelator). Representative images (left) and histogram for statistical analysis (right). Data were presented as mean ± S.D. (*n* = 3). *P < 0.05 *versus* AGEs‐BSA.

Mitochondrial damage triggers multiple pathways leading to NLRP3‐inflammasome activation 23. Here, we confirmed that AGEs‐BSA treatment promoted mitochondrial damage in human NP cells. As described in Figure 6A, a time‐dependent decrease in the ratio of red (JC‐1 aggregates) to green (JC‐1 monomers) coloration reflected a loss of mitochondrial membrane potential in NP cells treated with AGEs‐BSA. Compared with BSA control, AGEs‐BSA treatment decreased the red‐to‐green ratio after 48 hrs. Moreover, the difference in fluorescence intensity between cultured cells exposed to CoCl_2_ with (Tube 3) or without (Tube 4) ionomycin gradually narrowed over time, indicating the degree of mPTP activation (Fig. 6B). Following treatment of NP cells for 48 hrs, higher mPTP activation was detected in NP cells treated with AGEs‐BSA relative to that observed in control cells (Fig. 6B).

Furthermore, we found mutual interplay between calcium and ROS levels and mPTP activation in triggering mitochondrial damage. As shown in Figure 6C, treatment with BAPTA‐AM targeting intercellular calcium was capable of rescuing ROS generation, attenuating mPTP activation, and increasing mitochondrial membrane permeability. Additionally, decreases in mitochondrial membrane permeability and increases in mPTP were attenuated in the presence of the mitochondria‐targeted antioxidant MitoTEMPO. Similarly, in NP cells treated with the mPTP blocker cyclosporin A, the collapse of mitochondrial membrane permeability was prevented and mtROS was scavenged. The treatment with MitoTEMPO or cyclosporin A also attenuated calcium mobilization, which may have indicated that they formed a vicious circle enhancing NLRP3‐inflammasome activation.

### Rescued mitochondrial damage attenuated NLRP3‐inflammasome activation

**Figure 7:**
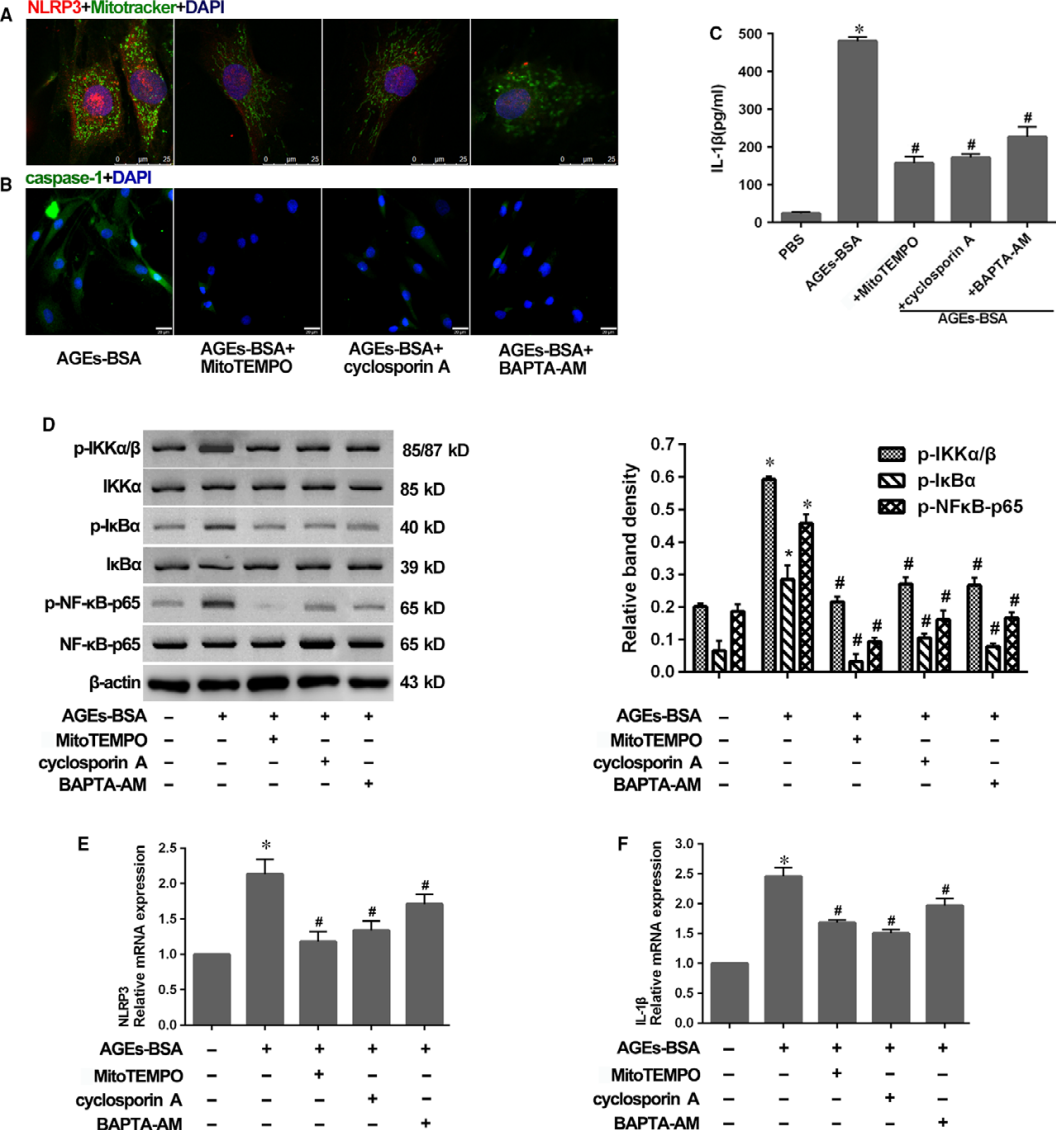
Rescued mitochondrial damage by MitoTEMPO, cyclosporin A or BAPTA‐AM attenuated NLRP3‐inflammasome activation. (**A**) Representative fluorescent images of co‐localization of NLRP3 and mitochondria in NP cells as described in Figure 6C. Red and green fluorescence indicated NLRP3 and mitochondria signals, respectively. Cell nuclei are stained by DAPI. (**B** and **C**) Representative fluorescent images of caspase‐1 activity (**B**) and enzyme‐linked immunosorbent assay for the secretion profiles of IL‐1β (**C**) in NP cells as described in Figure 6C. (**D**) Representative Western blot assay (left) and quantitation (right) showing phosphorylated (p‐) and total protein levels of IKKα/β, IκBα and NF‐κB‐p65, and (**E** and **F**) quantitative real‐time PCR for the transcript expression levels of NLRP3 and IL‐1β in NP cells as described in Figure 6C. Data were presented as mean ± S.D. (*n* = 3). *P < 0.05 *versus* control. #*P* < 0.05 *versus* AGEs‐BSA.

To investigate the role of mitochondrial damage in NLRP3‐inflammasome‐dependent inflammation induced by AGEs‐BSA treatment, human NP cells were pre‐treated with MitoTEMPO, cyclosporin A or BAPTA‐AM before AGEs‐BSA treatment. As expected, our results indicated that effects associated with pharmacological inactivation of the NLRP3 inflammasome involved attenuation of NLRP3 up‐regulation and prevention of co‐localization of NLRP3 with mitochondria (Fig. 7A). Furthermore, we observed the corresponding reduction in caspase‐1 activity (Fig. 7B) and IL‐1β secretion (Fig. 7C).

**Figure 8:**
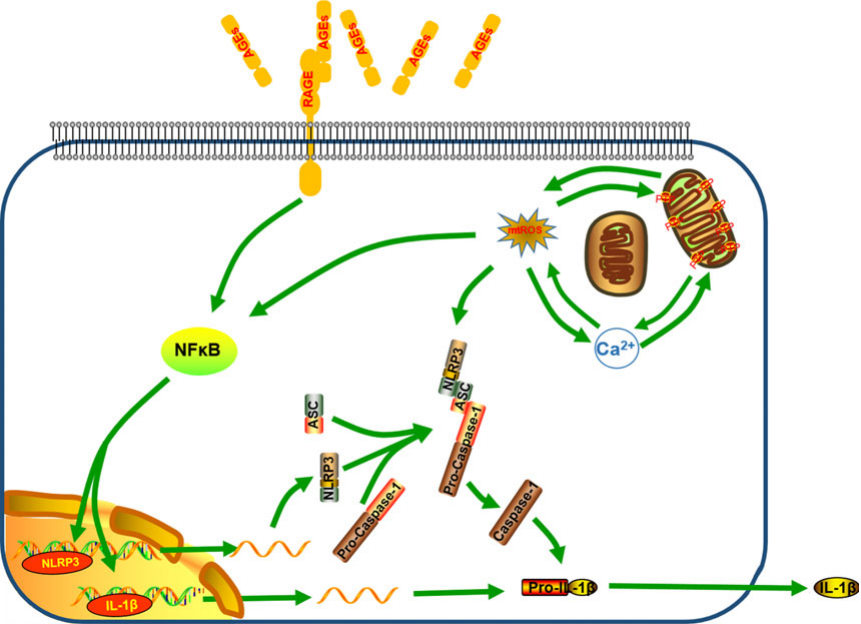
Schematic model illustrating how AGEs promoted NLRP3‐inflammasome activation. Receptor of advanced glycation end products (RAGE) and mitochondrial damage induced by advanced glycation end products (AGEs) promoted the NF‐κB activation and secondary the transcription of NLRP3 and IL‐1β. Moreover, Mitochondrial damage enhanced the assembly of NLRP3 inflammasome, then the cleavage of pro‐caspase‐1 and pro‐IL‐1β.

Moreover, according to the attenuation of NLRP3 up‐regulation as above, we determined whether mitochondrial damage influenced NF‐κB activation. As expected, we observed reduced NF‐κB activation following treatment with MitoTEMPO, cyclosporin A or BAPTA‐AM (Fig. 7D), and subsequently rescued NLRP3 and pro‐IL‐1β expression in response to AGEs‐BSA treatment (Fig. 7E and F). It indicated that RAGE and mitochondrial damage primed NLRP3 and pro‐IL‐1β activation as upstream signals of NF‐κB activity, whereas mitochondrial damage was critical for the assembly of inflammasome components (Fig. 8).

## Discussion

Both AGEs and inflammation play critical roles in the pathophysiology of IVD degeneration 1, 15, 16. However, the mechanism by which AGEs stimulate the inflammatory response in the IVD remains to be elucidated. Here, we demonstrated that AGEs‐BSA treatment promoted an inflammatory response in NP cells *via* NLRP3‐inflammasome activation. A key observation was that NLRP3‐inflammasome components were up‐regulated in NP tissues from patients with DDD, as well as in human NP cells stimulated *in vitro* with AGEs‐BSA. NLRP3 knockdown or inhibition of caspase‐1 and IL‐1β reduced IL‐1β generation and attenuated anabolic and catabolic activities induced by AGEs‐BSA. Furthermore, RAGE/NF‐κB and mitochondrial damage by calcium, mtROS and mPTP contributed to NLRP3‐inflammasome activation.

A growing body of evidence indicates that endogenous molecules from cell metabolism or degradation of the extracellular matrix trigger inflammation, acting as ‘danger signals’ called damage‐associated molecular patterns (DAMPs) 13. Degraded fragments of proteoglycans, glycosaminoglycans and collagens, which are otherwise essential components of the IVD, accumulate and induce tissue degeneration and inflammation as human beings age 9, 24, 25, 26. Similarly, AGEs accumulate in the IVD and elicit degeneration 15, 16, 17. Therefore, we proposed that AGEs in the IVD represent a class of DAMPs that activate the NLRP3 inflammasome in a manner reminiscent of other chronic inflammatory diseases. In line with this hypothesis, our results demonstrated that AGE disposition and NLRP3‐inflammasome activation correlated with IVD degeneration. Furthermore, interference of the NLRP3 inflammasome rescued degenerative phenotype of NP cells induced by AGEs‐BSA treatment *in vitro*. Overall, our data indicated that the NLRP3 inflammasome may contribute to AGEs‐induced inflammation and degeneration in IVD.

Here, we determined that the NLRP3 inflammasome contributes to AGE‐induced inflammation. The canonical model of NLRP3 activation requires an initial priming signal that up‐regulates transcription of NLRP3 and pro‐IL‐1β, followed by a second activation signal that assembles the NLRP3 inflammasome and enhances maturation of pro‐caspase‐1 and pro‐IL‐1β 27. However, the molecular mechanism of NLRP3‐inflammasome activation in this process remains to be clarified. Previous studies identified pattern‐recognition receptors, such as Toll‐like receptor (TLR)2 and TLR4, which mediate inflammation and IVD degeneration in response to pathogens and tissue damage in NP cells 9, 28. RAGE is also a multi‐ligand transmembrane receptor, with AGEs identified as primary ligands. More specifically, RAGE and its intercellular signalling pathway are related to IVD degeneration 15, 18. In this study, we investigated the role of RAGE in AGEs‐induced NLRP3‐inflammasome activation. Previous studies identified that RAGE relays intracellular signals *via* the NF‐κB signal transduction pathway 29, which is also critical for up‐regulating the transcription of both NLRP3 and pro‐IL‐1β 30. Through activation and inhibition experiments in human NP cells, we confirmed the essential role of the RAGE/NF‐κB pathway in AGEs‐induced NLRP3‐inflammasome activation by promoting the NLRP3 and pro‐IL‐1β expression.

Several models have been proposed for NLRP3‐inflammasome activation, while the models of ROS generation and calcium mobilization are more likely to link to others and all NLRP3 agonists trigger ROS generation and calcium mobilization 20, 21. Intriguingly, Naser and colleagues have found that AGEs could lead to the disorder of calcium signalling, in which ROS generation was involved 31. Based on this evidence, we proposed that ROS generation and calcium mobilization induced by AGEs resulted in the inflammatory response in NP cells through the activation of the NLRP3 inflammasome. We found a demonstrable increased ROS and calcium level in NP cells under the treatment of AGEs‐BSA. The source of ROS is of variety, but mitochondria are assumed as the main site of intracellular ROS generation. Notably, mtROS was confirmed to enhance multiple activities of NP cells involving autophagy, apoptosis and catabolic metabolism 32, 33, 34. Similarly, NP cells showed a marked increase in mtROS level, and a specific mtROS scavenger, MitoTEMPO, could reduce the ROS level. Then, MitoTEMPO and BAPTA‐AM efficiently reduced the caspase‐1 activity and IL‐1β secretion.

ROS and calcium, as major secondary messengers, conducted multiple signalling pathways within cells and showed close contact. Mitochondria, acting as a complex decoding station, manipulate ROS and calcium haemostasis and transfer signallings 35, 36. Intriguingly, with respect to NLRP3‐inflammasome activation, increasing endogenous DAMPs were identified as involved in mechanisms associated with mitochondrial damage 22, 37, 38, including the secondary redox and calcium disorder, while, consequently, mitochondria also are the target of oxidative stress and calcium overload 21, 39, 40. How do they interact with each other and play together? The mPTP plays a pivotal role in regulating mitochondrial function. Transient opening of the mPTP is critical for ATP synthesis and calcium and ROS signalling, while prolonged opening can result in the rupture of the outer mitochondria membrane, collapse of mitochondrial membrane potential and depletion of cellular ATP, mitochondrial calcium efflux and ROS production 41, 42. Rotenone‐induced mtROS could promote mPTP formation and opening 43, and mtROS scavenging by SOD1 and MitoTEMPO attenuated mPTP opening 39. Similarly, mitochondrial calcium is the master regulator of mPTP opening, and its over‐uptake is critical for mPTP opening and mitochondrial damage 41. Here, marked mitochondrial damage occurred in human NP cells following AGEs‐BSA treatment, concomitant with increased mPTP activation. Moreover, we analysed the mutual association of mtROS, calcium and mPTP converging on mitochondria function. As shown, there appears to be a vicious circle among mtROS, calcium and mPTP, centred on the mitochondria under the treatment of AGEs‐BSA.

Previous studies demonstrated that neither priming nor an activation signal alone can significantly initiate NLRP3 activity, although it is unclear how both signals synergize. One recent study has approved that ROS, deriving from H_2_O_2_ and rotenone, promoted the priming of NLRP3‐inflammasome activation 44. Two‐step deubiquitination was proposed to activate NLRP3 in response to mtROS 45. Here, we directly analysed the NF‐κB signalling under the integration of mtROS, calcium and mPTP. We found that inhibition of their disorders attenuated NF‐κB activation induced by AGEs‐BSA. Previous studies also reported various ways in which oxidative stress influences NF‐κB activation 46; however, the detailed mechanisms of mtROS, calcium and mPTP involved in NF‐κB activation require further studies.

In summary, we demonstrated that AGE accumulation and NLRP3‐inflammasome activation in the NP are associated with IVD degeneration. Additionally, *in vitro* studies showed that AGEs act as endogenous DAMPs promoting mitochondrial damage and inducing an inflammatory response in NP cells *via* the NLRP3‐inflammasome. Our findings suggested that accumulation of AGEs in the NP may drive inflammation‐related degeneration of the IVD. These results may aid in the identification of new therapeutic strategies.

## Conflicts of interest

The authors confirm that there are no conflict of interests.
